# Effect of different salt concentrations on the gamma‐aminobutyric‐acid content and glutamate decarboxylase activity in germinated sorghum (*Sorghum bicolor* L. Moench) grain

**DOI:** 10.1002/fsn3.2821

**Published:** 2022-03-10

**Authors:** Maha F. Elbaloula, Amro B. Hassan

**Affiliations:** ^1^ Department of Food Science and Technology College of Agricultural Studies Sudan University of Science and Technology Khartoum Sudan; ^2^ Department of Food Science and Nutrition Faculty of Food and Agricultural Sciences King Saud University Riyadh Saudi Arabia; ^3^ Environment and Natural Resource Desertification Research Institute (ENDRI) National Center for Research Khartoum Sudan

**Keywords:** GAD activity, gamma‐aminobutyric‐acid, germination, salt concentration, sorghum

## Abstract

This study aimed to estimate the γ‐aminobutyric acid (GABA) content and glutamate decarboxylase activity (GAD) in germinated sorghum grain as affected by different concentrations of NaCl, pyridoxal 5‐phosphate (PLP), and CaCl_2_. In general, the obtained results revealed that the addition of low doses of NaCl (40 mmol/L), PLP (90 mmol/L), and CaCl_2_ (0.5 mmol/L) to the germination culture significantly (*p* < .05) enhanced the GABA content and subsequently improved the GAD activity in sorghum grains. Moreover, CaCl_2_ played a dominant role in the extent of enzymolysis, followed by NaCl and PLP. Regarding the GABA content, the optimal concentration of the NaCl, PLP, and CaCl_2_ was estimated as 41.07 mmol/L, 82.62 μmol/L, and 0.40 mmol/L, respectively. Under this optimal culture medium, the maximum GABA content was 0.336 mg/g. In conclusion, the findings of this work would provide a scientific basis for the industrialized production of GABA‐enriched sorghum foods.

## Graphical abstract

Different concentrations of NaCl, PLP, and CaCl_2_ were added to germination media. The GAD activity and GABA content of germinating sorghum grains were investigated. The addition of low doses of salt improves GAD activity and GABA content in sorghum grains.

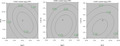

## 1 INTRODUCTION

Sorghum (*Sorghum bicolor* L. Moench) is one of the important cereal crops in developed and developing countries with many unique characteristics. It has received more attention recently as a functional crop for biofuel production and animal feed (Przybylska‐Balcerek et al., 2020). It is also considered a significant source of carbohydrates, protein, minerals, antioxidants, vitamins, and a gluten‐free diet for the poor people of Africa and Asia (Ratnavathi et al., 2016). However, sorghum grains have been processed to improve their amount of nutrients and phytochemicals. Sorghum is traditionally fermented or germinated before consumption (Almaiman et al., 2021).

Germination is common in the sorghum processing industry to enhance the inner hydrolytic enzyme activity and modify its grain structure and components. During germination, several significant changes occur, including the interconversion and production of new compounds. It increases the content of cereals' functional parts and enhances their healthful functions. Moreover, it enhances the capability of hydroxyl radical inhibition in grains (Bai et al., 2008; Li et al., 2007). In particular, AL‐Quraan et al. (2019) stated that the functional component of many of the cereal grains, particularly γ‐aminobutyric acid content (GABA), as well as the activity of the glutamate decarboxylase (GAD), was increased after the germination process. It was stated that both germination temperature and time, particularly at 25 C for 3 days, influenced the accumulation of GABA in sorghum (Garzon & Drago, 2018). Additionally, Garzón et al. (2019) found that the GABA content increased during the brewing processing of white sorghum. Increasing GBA content in grains might be due to the seed stress conditions as stated by Garzón et al. (2016).

In the human and animal bodies, the γ‐aminobutyric acid (GABA) was represented by a series of health functions. It is also considered an inhibitory neurotransmitter in the brain and spinal cord of mammals (Komatsuzaki et al., 2007). According to Bouché and Fromm (2004), GABA is mostly formed over the decarboxylation of glutamic acid catalyzed by the enzyme glutamate decarboxylase (GAD, EC4.1.1.15). Additionally, it has been stated that several characteristics and functions of GABA were highly associated with the increase of GAD activity. These functions were inspired by GAD activity resulting from various environmental stresses, such as anoxia, water stress, decreasing cellular pH, temperature change, and mechanical stress (Kinnersley & Turano, 2000). Komatsuzaki et al. (2007) found that the GABA content in germinated brown rice was sharply increased compared to raw rice samples.

It was noted that GABA accumulation usually parallels GAD activity's stimulation. Therefore, the increase in the concentration of cytosolic H^+^ could enhance GAD activity. Furthermore, Shelp et al. (1999) stated that the pyridoxal‐5‐phosphate (PLP) enhanced the GAD activity in plant tissues. Moreover, the activity of GAD in plants is motivated by the Ca^2+^/calmodulin messenger system (Baum et al., 1993). Hence, enhancing the GAD activity in sorghum grains might undoubtedly increase the GABA synthesis.

As far as we know, there are no reports about sorghum GABA and GAD activity, neither in sorghum raw materials nor in germinated sorghum. Hence, this study was carried out to estimate the GABA and the GAD accumulation in germinated sorghum grain as an effect of NaCl, PLP, and CaCl_2_ concentration.

## 2 MATERIALS AND METHODS

### 2.1 Materials

Sorghum grains cultivar (Butanna) was obtained from the Arab‐Sudanese seed company, Khartoum, Sudan. The materials were carefully cleaned and stored in polythene bags at 4°C before use. GABA, PLP, and phenylisothiocyanate (PITC) were obtained from Sigma Chemicals Co. Acetonitrile was of high‐performance liquid chromatography (HPLC) grade. All other chemicals and reagents used were of analytical grade.

### 2.2 Experimental design and sorghum germination

To evaluate the effect of salts concentrations on the GABA content and GAD activity in germinated sorghum, grains were germinated (at 30°C) in germination cultures with different concentrations of NaCl, PLP, and CaCl_2_ 30–70 mmol/L, 0–150 μmol/L, and 0–10 mmol/L, respectively. All solutions were prepared using 0.01 mol/L citrate phosphate buffer solution (pH 6.0). Before germination, sorghum grains were steeped in deionized water for 6 h, surface‐sterilized with 1.0% (v/v) NaClO at 25 ± 2°C for 30 min and then dipped three times with deionized water.

Germination was carried out in a culture plat as Yang et al. (2016) described. Firstly, the sorghum seeds were surface‐sterilized with NaClO (1.0%) for 30 min. Then sorghum grains were placed in a culture plate with different concentrations and incubated in the dark for 3 days at 32 ± 2°C.

### 2.3 Determination of GABA content in germinated sorghum

The content of the GABA was determined according to the method described by Bai et al. (2009) with slight modifications. One gram of germinated sorghum flour was mulled with 6 ml of 4% acetic acid for 1 h to extract the GABA followed by centrifugation at 6000 g for 15 min. Then 4 ml of ethanol was added to the supernatant to remove macro‐molecular polymers. The purified supernatant was evaporated (0.1 MPa, 45°C) to volatilize the acetum acid and ethanol. The residues were dissolved with 0.5 ml of distilled water and centrifuged at 4000 g for 10 min. The purified and filtered supernatant were analyzed by HPLC (Agilent 1200) as Sawai et al. (2001) described.

### 2.4 Determination of GAD activity in germinated sorghum

The GAD activity of the germinated sorghum was measured according to Zhang et al. (2007) method. One gram of germinated sorghum was mixed with 5 ml of potassium phosphate buffer (1/15 mol/L, pH 5.8; 2 mmol/L β‐mercaptoethanol, 2 mmol/L, EDTA, and 0.2 mmol/L PLP) and then centrifuged at 10,000 g for 15 min at 4°C. A mixture of 200 μl of the supernatant and 100 μl of the substrate (1% of Glu, pH 5.8) was incubated firstly at 40°C for 2 h and then at 90°C for 5 min. The centrifugal suspension was filtered through a 0.45 μm membrane filter. The filtrate was analyzed for GABA content as mentioned above. GAD enzyme activity was calculated as the release of 1 μmol of GABA produced per 1 h at 40°C.

### 2.5 Optimization of components for GABA accumulation

After determining the preliminary range of NaCl, PLP, and CaCl_2_ through a single‐factor test, a response surface methodology (RSM) with three‐level‐three‐factor based on the Box‐Behnken design (BBD) was employed in the optimization study. The NaCl (*X*_1_), PLP (*X*_2_), and CaCl_2_ (*X*_3_) were the independent variables selected to be optimized for GABA accumulation (Table 1). The GABA content (*Y*) represented the grouping of the independent variables. To minimize the systematic errors due to inexplicable variability in the experimental responses, all the experiments were conducted in random mode. The following quadratic equation explained the behavior of the system:

(1) $$Y=\beta_{0}+\sum \beta_{i}X_{i}+\sum \beta_{\text{ii}}X_{i}^{2}+\sum \beta_{\text{ij}}X_{i}X_{j}$$

where *Y* is the dependent variable. *β*_0_ is an offset term. *β*_i_, *β*_ii_, and *β*_ij_ are the linear, quadratic, and interaction regression coefficients, respectively and *X*_i_ and *X*_j_ are levels of the independent variables.

**TABLE 1:** Analytical factors and levels for RSM, and results of response surface analysis

Independent variables	Levels		
	−1	0	1
*X*_1_: NaCl (mmol/L)	30.00	40.00	50.00
*X*_2_: PLP (μmol/L)	60.00	90.00	120.00
*X*_3_: CaCl_2_ (mmol/L)	0.20	0.50	0.80

No.	*X*_1_ NaCl (mmol/L)	*X*_2_ PLP (μmol/L)	*X*_3_ CaCl_2_ (mmol/L)	*Y*: GABA content (mg/g DW)
1	−1	−1	0	0.255
2	1	−1	0	0.317
3	−1	1	0	0.292
4	1	1	0	0.281
5	−1	0	−1	0.300
6	1	0	−1	0.285
7	−1	0	1	0.263
8	1	0	1	0.287
9	0	−1	−1	0.327
10	0	1	−1	0.293
11	0	−1	1	0.258
12	0	1	1	0.284
13	0	0	0	0.331
14	0	0	0	0.334
15	0	0	0	0.335

To evaluate the RSM of the independent variables, the experimental design and calculation of the data were estimated using the Design‐Expert 8.0.5 (Trial Version, State‐Ease Inc.) software. Experimental data were expressed as the mean ±standard deviation with three replications.

## 3 RESULTS AND DISCUSSION

### 3.1 Effects of NaCl concentration on GABA content and GAD activity of germinated sorghum

**FIGURE 1:**
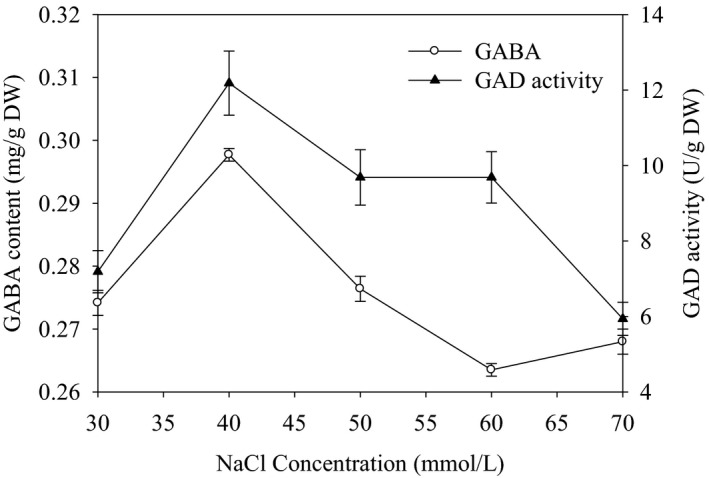
Effects of NaCl addition on GAD activity and GABA content in germinated sorghum

Figure 1 represents the GABA content and GAD activity in germinated sorghum grain as influenced by different NaCl concentrations. The figure shows that the GABA content was less than 0.28 mg/g D.W. at the lowest NaCl concentration 30 mmol/L. In contrast, the highest GABA content (0.308 mg/g D.W.) was observed at the concentration of 40 mmol/L. As the concentration of the NaCl increased to more than 40 mmol/L, the GABA content was sharply decreased to 0.295, 0.295, and 0.263 mg/g D.W. for the concentrations of 50, 60, and 70 mmol/L, respectively.

A similar trend was also observed in the activity of GAD in the germinated sorghum as influenced by different NaCl concentrations. Concentration up to 40 mmol/L showed higher GAD activity (10.05 U/g). However, it was found significantly low at 60 and 70 mmol/L (Figure 1). According to the obtained results, 40 mmol/L NaCl addition was most favorable for GABA accumulation and GAD activity. A similar observation was also reported by Wang et al. (2017), who stated that a high concentration of NaCl decreased the endogenous GABA concentration and decreased glutamate decarboxylase (GAD) activity. It was noted that the GAD activity usually generates more GABA formation. So salt stress caused by NaCl application was strongly reported to stimulate GAD activity in maize (Wang et al., 2017) and thus accumulate GABA in plants as an adaptive response to the stress condition. Recently, Wang et al. (2021) found that low concentration of NaCl could stimulate the endogenous GABA synthesis and regulate GABA metabolism during the germination of barley.

### 3.2 Effects of PLP concentration on GABA content and GAD activity of germinated sorghum

**FIGURE 2:**
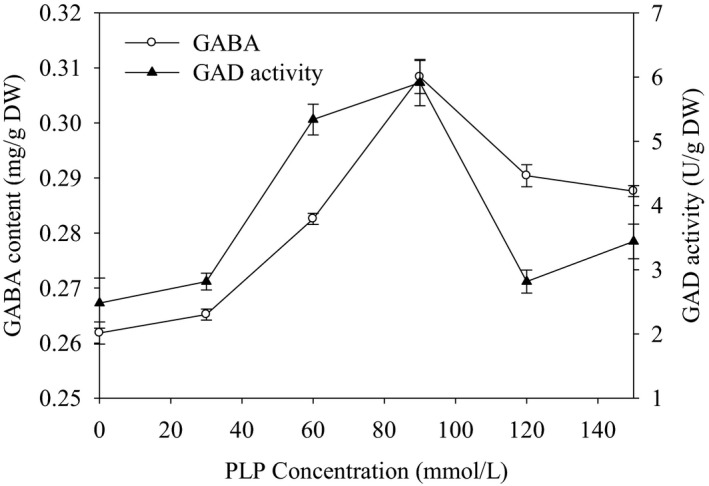
Effects of PLP addition on GAD activity and GABA content in germinated sorghum

The GABA content and the GAD activity in germinated sorghum as influenced by the addition of different PLP concentrations was described in Figure 2. In the control sample, the GABA content was found to be 0.262 mg/g, while the GAD activity was estimated as 2.5 U/g. It was observed that the addition of the PLP significantly (*p* < .05) enhanced GAD activity and GABA yield compared to the control (Figure 2). Moreover, the highest GAD activity and the highest GABA content were obtained at the addition of 90 μmol/L PLP to the culture medium. This might be because PLP plays an imperative role in encouraging GAD activity as an enzyme cofactor (Shelp et al., 1999). So, adding PLP to the culture medium would affect GABA production. Likewise, Komatsuzaki et al. (2005) reported that GABA content was increased by the addition of PLP to the medium during the germination process.

Moreover, Yang et al. (2011) found that the GAD activity was increased germinated in Fava Bean (*Vicia faba* L.) seeds due to the addition of PLP to the germination medium. In general, our findings revealed that the addition of PLP enhanced both the GABA content and GAD activity in germinated sorghum. However, the appropriate PLP concentration should be noted, and 90 μmol/L was optimal in the present experiment.

### 3.3 Effects of CaCl_2_ concentration on GABA content and GAD activity of germinated sorghum

**FIGURE 3:**
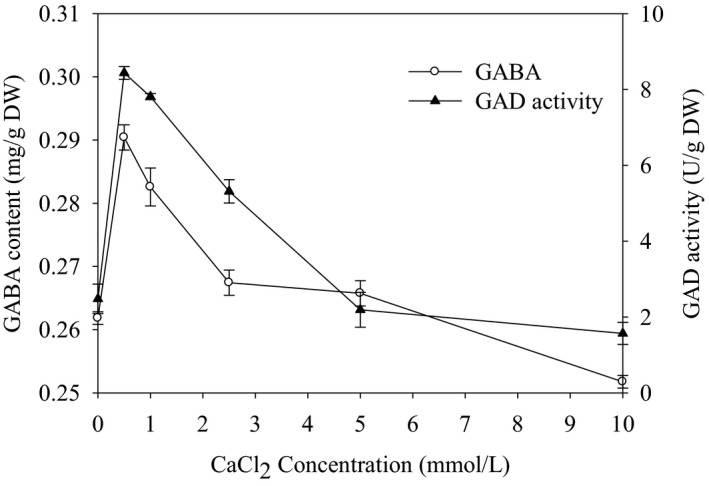
Effects of CaCl_2_ addition on GAD activity and GABA content in germinated sorghum

The effects of CaCl_2_ addition on GABA content and GAD activity in germinated sorghum were presented in Figure 3. It was found that both the GABA content and GAD activity were sharply increased with the addition of low CaCl_2_ concentration (>1 mmol/L). The maximum content of GABA (0.3 mg/g) was found at 0.50 mmol/L CaCl_2_, where the GAD activity was also the highest at the same CaCl_2_ concentration. A higher concentration of CaCl_2_ 10 mmol/L caused a drastic reduction of GABA yield and GAD activity. Our findings also indicated that adding Ca^2+^ motivated GAD activity and encouraged GABA production in germinated sorghum. Smith et al. (2010) showed that Ca^2+^ is very important for activating GAD activity in plants. These findings also revealed that a Ca^2+^/CaM signal transduction pathway controlled GAD. It has been stated that the increase of GABA is intensely encouraged by salt stress (Kim et al., 2007). Likewise, Yang et al. (2015) found that the content of GABA and the GAD activity in cotyledon and shoot of germinated fava bean increased after adding CaCl_2_.

### 3.4 Statistical analysis and the model fitting

A total of 15‐run BBD with three factors and three levels was used to fit a second‐order response surface to illustrate the interactive effects of NaCl, PLP, and CaCl_2_ concentrations on GABA content. Table 1 lists the process variables and experimental data. Table 2 shows the results of the analysis of variance, goodness‐of‐fit, and the models' adequacy. The maximum value (0.336 mg/g DW) was found at NaCl 41.07 mmol/L, PLP 82.63 μmol/L, and CaCl_2_ 0.40 mmol/L. The parameters of the following Equation (2) were obtained by multiple regression analysis of the experimental data:

(2) $$Y=\;0.33‐\left(7.500E‐003\right)X_{1}‐\left(8.750E‐004\right)X_{2}‐0.014X_{3}‐0.018X_{1}X_{2}+\left(9.750E‐003\right)X_{1}X_{3}+0.015X_{2}X_{3}‐0.027X_{1}^{2}‐0.020X_{2}^{2}‐0.023X_{3}^{2}$$

**TABLE 2:** Statistical ANOVA for regression model of response surface methodology for optimizing salt concentrations of γ‐aminobutyric acid (GABA) accumulation

Variables	Sum of squares	*df*	Mean square	*F* value	*p* value prob >F	Significance^a^
Model	9.947E−003	9	1.105E−003	11.75	.0072	**
*X*_1_ *‐NaCl*	4.500E−004	1	4.500E−004	4.78	.0804	
*X*_2_ *‐PLP*	6.125E−006	1	6.125E−006	0.065	.8088	
*X*_3_ *‐CaCl_2_*	1.596E−003	1	1.596E−003	16.97	.0092	**
*X*_1_ *X*_2_	1.332E−003	1	1.332E−003	14.16	.0131	*
*X*_1_ *X*_3_	3.802E−004	1	3.802E−004	4.04	.1006	
*X*_2_ *X*_3_	9.000E−004	1	9.000E−004	9.57	.0271	*
*X*_1_^2^	2.675E−003	1	2.675E−003	28.43	.0031	**
*X*_2_^2^	1.502E−003	1	1.502E−003	15.96	.0104	*
*X*_3_^2^	1.897E−003	1	1.897E−003	20.16	.0065	*
Residual	4.704E−004	5	9.408E−005			
Lack of fit	4.618E−004	3	1.539E−004	35.52	.0275	*
Pure error	8.667E−006	2	4.333E−006			
Cor total	0.010	14				
	*R* ^2^ = .9548	*R*_adj_^2^ = .8736	CV = 3.28%			

^a^ *means significant at *p* < .05, **means significant at *p* < .01.

The fit of the predictive model was checked by the determination of the coefficient *R* ^2^, which was .9548, indicating that the model equation could not explain only 4.52% of the total variations. Furthermore, the adjusted determination coefficient (*R*_adj_^2^ = .8736) also displayed that the model was highly significant. Simultaneously, a meager value of 3.28% of the coefficient of the variation (CV) indicated a remarkably high degree of precision and a good deal of reliability of the obtained findings. Finally, the model *p*‐value (prob >F) was .0072, suggesting that the model was significant.

The *t*‐test and *p* values were applied to recognize the outcome of each factor on GABA content. The *p* values were performed as a tool to check the significance of each coefficient. The smaller value was, the more significant the corresponding coefficient (Guo et al., 2010). As shown in Table 3, the linear effects of CaCl_2_ were significant (*p* < .01) for GABA accumulation. Furthermore, the interactive terms between NaCl and PLP (*p* < .05), PLP and CaCl_2_ (*p* < .01) were significant model terms for GABA accumulation. The quadratic term of NaCl was significant at *p* < .01, while that of PLP and CaCl_2_ was significant at *p* < .05. Results showed that among the three independent variables, CaCl_2_ played a dominant role in the extent of enzymolysis, followed by NaCl and PLP.

**TABLE 3:** Optimizing and results of validation trials

	NaCl (mmol/L)	PLP (μmol/L)	CaCl_2_ (mmol/L)	GABA content (mg/g DW)	
				Observed value	Predicted value
Optimal conditions	41.07	82.63	0.40	0.332 ± 0.003	0.336

### 3.5 Optimization of components for GABA content and model verification

**FIGURE 4:**
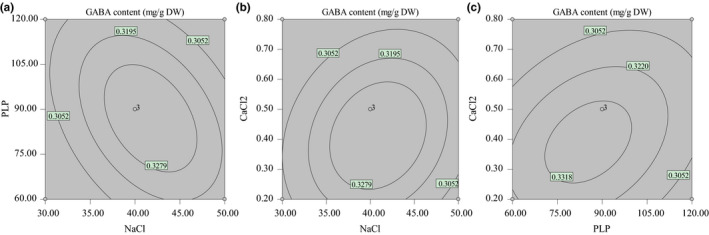
(a) Response surface contour plot showing the interactions between NaCl and PLP. (b) Response surface contour plot showing the interactions between NaCl and CaCl_2_. (c) Response surface contour plot showing the interactions between PLP and CaCl_2_

The optimal levels of each variable for maximum GABA content were estimated. The response surface plots were determined by plotting the response vs. any two independent variables, while the other one was fixed at zero levels. The results of GABA content affected by the interactions of variables are illustrated in Figure 4a,b,c.

The contour plot in Figure 4a, which gives the CaCl_2_ addition at zero levels (0.50 mmol/L), shows that GABA content increased firstly and then decreased with NaCl concentration from 30 to 50 mmol/L. The same changing trend was also observed with increasing PLP concentration from 60 to 120 μmol/L. Figure 4b presents the contour plot at varying NaCl and CaCl_2_ addition at fixed PLP content (zero levels). It can be seen that both increasing NaCl and CaCl_2_ concentration led to a trend of increasing firstly and then decreasing in the GABA content, which corresponded with that of the single factor experiment. The above results were further confirmed in Figure 4c where NaCl concentration was fixed at zero levels (40 mmol/L), showing that GABA content increased and decreased afterward with increasing PLP and CaCl_2_ range.

According to the single factor study (Figure 4a,b,c), the optimal culture modules for GABA content were 41.07 mmol/L NaCl, 82.63μmol/L PLP, and 0.40 mmol/L CaCl_2_ in the culture solution. Under this optimal culture medium, the maximum GABA content was 0.336 mg/g D.W. Verifying the model equation was tested using the selected optimal culture solution. The maximum predicted GABA content and experimental content were given in Table 3. The observed value was 0.332 ± 0.003 mg/g D.W., which was well suited to the value predicted by the model and no significant differences (*p* > .05) were observed. The results proved that the model was valid and practical.

## 4 CONCLUSION

In this study, the effect of different concentrations of the NaCl, PLP, and CaCl_2_ on the GABA content and GAD activity in germinated sorghum grain was investigated. The finding revealed that the addition of NaCl, PLP, and CaCl_2_ significantly increased the GABA content and also caused a further enhancement in the GAD activity of germinated sorghum. However, high doses of these salts caused a reduction in the GAB content and GAD activity. Moreover, among the three independent variables, CaCl_2_ played a dominant role in the extent of enzymolysis, followed by NaCl and PLP. Generally, the addition of low concentrations of NaCl, PLP, and CaCl_2_ during the germination medium of sorghum increased its GABA. In addition, it enhanced GAD activity which would offer GABA‐enriched sorghum foods for food manufacturing.

## CONFLICT OF INTEREST

The authors declare that there is no conflict of interest.

## ETHICAL APPROVAL

This work does not involve any human or animal experiments.

## Data Availability

The data used to support the findings of this study are included in the article and are available from the corresponding author upon reasonable request.
